# Synthesis of Planar-Type ZnO Powder in Non-Nano Scale Dimension and Its Application in Ultraviolet Protection Cosmetics

**DOI:** 10.3390/ma16052099

**Published:** 2023-03-05

**Authors:** Jung-Hwan Lee, Gun-Sub Lee, Eung-Nam Park, Dong-Hyeon Jo, So-Won Kim, Hee-Chul Lee

**Affiliations:** 1 Energy Business Unit, Duckjin Co., 341, Gongdan 1-Daero, Siheung 15078, Republic of Korea; 2 Department of Advanced Materials Engineering, Tech University of Korea, Siheung 15073, Republic of Korea

**Keywords:** zinc oxide powder, non-nanosized particle, planar-type, UV protection, cosmetic formulation

## Abstract

ZnO is one of the most widely used inorganic sunscreens, owing to its fine particle size and UV light shielding capability. However, powders at nanosizes can be toxic and cause adverse effects. The development of non-nanosized particles has been slow. The present work investigated synthesis methods of non-nanosized ZnO particles for ultraviolet protection application. By altering the starting material, KOH concentration, and input speed, the ZnO particles can be obtained in different forms, including needle type, planar type, and vertical wall type. Cosmetic samples were made by mixing different ratios of synthesized powders. The physical properties and the UV blockage efficacy of different samples were evaluated using scanning electron microscopy (SEM), X-ray diffraction (XRD), particle size analyzer (PSA), and ultraviolet/visible (UV/Vis) spectrometer. The samples with 1:1 ratio of needle-type ZnO and vertical wall-type ZnO exhibited superior light blocking effect owing to improved dispersibility and prevention of particle agglomeration. The 1:1 mixed sample also complied with the European nanomaterials regulation due to the absence of nanosized particles. With superior UV protection in the UVA and UVB regions, the 1:1 mixed powder showed potential to be used as a main ingredient in UV protection cosmetics.

## 1. Introduction

Due to recent climate changes, human bodies are more likely to be overexposed to ultraviolet light than in the past, and more consumers are recognizing the risks of ultraviolet (UV) light overexposure, such as early aging of skin and skin cancers. Ultraviolet protection cosmetics (i.e., sunscreens), functional cosmetics, can be broadly divided into organic and inorganic sunscreens. Organic sunscreens, which utilize organic molecules to chemically absorb UV light to prevent penetration of UV light into the skin, has been used for a long time, but has disadvantages such as skin toxicity, liability to trigger allergic reactions, and discolorations. The need to overcome the limitations of organic sunscreens has motivated research into inorganic sunscreens to [1,2,3]. Inorganic sunscreens block ultraviolet light by primarily absorbing and diffracting ultraviolet light; metal oxides with high refractive index such as TiO_2_, CeO_2_, and ZnO are commonly used [4,5]. Among them, ZnO, which has been attracting attention for its broad range of applications in many areas, including gas sensors, biosensors, solar cells, and electrochemical cells, is more widely used as inorganic sunscreens owing to its fine particle size, non-toxicity, and UV light shielding capability of UVB (280–320 nm) and UVA (320–400 nm) that reflects from the ground and harms skin [6,7,8]. To prevent heavy white residue and improve the sun protection factor, studies have developed ZnO in powder form with variously shaped molecules at 30–50 nm sizes to improve its light blocking rate [9,10]. However, recent studies indicated that powders at nanosizes can be more toxic and easily penetrate into the human body, causing adverse effects [11,12,13]. Therefore, there has been a decrease in the use of sunscreens with nanosized particles. Extensive research has been conducted on non-nano ZnO synthesis that complies with nanomaterial regulations. Most of the synthesized non-nanosized ZnO sunscreens have secondary particles (resulting from agglomeration of primary particles with sizes of 1–100 nm) in sizes of more than 100 nm [14,15]. In practice, the development of true non-nanosized particles that can lower the skin penetration has been slow. In the previous research, our group also independently developed a planar-shaped ZnO powder with a high aspect ratio of non-nano particle size in two directions for UV blocking. Moreover, when mixed with TiO_2_, this powder showed relatively good UV protection in a wide range of wavelengths and formulation stability in cosmetic formulations [16].

Generally, various methods such as sol-gel [17,18,19], chemical precipitation [20,21], and solvothermal and hydrothermal synthesis [22,23] have been developed to synthesize ZnO particles with uniform morphology and size. However, there has yet to be clear research on the shape and size control of the powder, so research on this is necessary.

In this study, we obtained ZnO powder using a wet precipitation method in which a base is added to an acid solution to neutralize it. We first synthesize ZnO powders with various shapes, including needle type, planar type, and vertical wall type by altering starting material, KOH concentration and input speed, and the size and clustering were controlled to compare the characteristics with commercial nanosized ZnO powder. Next, sun blockage performance of different types of ZnO powders and powder mixtures were investigated. Based on the analysis results, the developed powders were evaluated for their suitability as raw materials for sunscreen cosmetics.

## 2. Materials and Methods

### 2.1. Synthesis and Characterization of ZnO Powder

**Figure 1:**
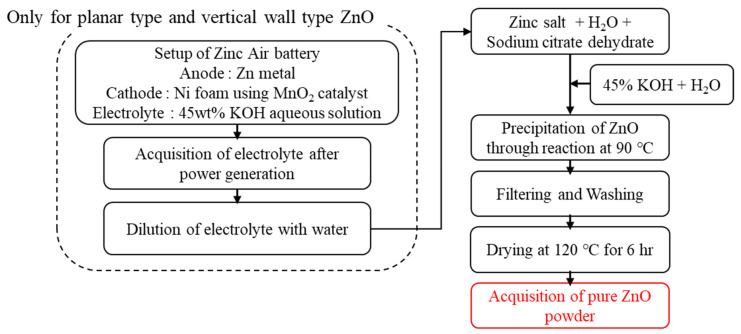
Diagram illustrating fabrication of ZnO powder.

To synthesize ZnO powders with different morphologies, the starting materials and synthesis conditions were adjusted as shown in Figure 1 and Table 1. The starting materials of 1.5 M zinc acetate (Jiangsu Kolod Food Ingredients Co., Lianyungang, China, 99.85%), or 1.5 M zinc chloride (Samchun, Seoul, Republic of Korea, 98%) and 0.18 mM sodium citrate (Daejung, Siheung, Republic of Korea, 99%) were put in the batch reactor in the base and stirred with distilled water at 90 °C for 2 h.

For synthesizing planar-type and vertical wall-type ZnO, electrolytes recovered from zinc–air battery power generation system were utilized in order to synthesize ZnO with high aspect ratios by suppressing the vertical growth on top of the [0001] face, which has high polarity due to the effect of Zn(OH_4_)^2−^ anion inside the electrolyte [16]. Then, various concentrations of aqueous solutions made by diluting 45% KOH in distilled water were added, and the input speed was altered until the pH reached a point where the purity of each batch reactor was maximized. After the reaction was completed, the mixture was stirred and aged for 2 h at 70 °C. After leaving the mixture to stand for 12 h, the precipitates were filtered using a fine filter, cleaned thoroughly with distilled water, and dried at 120 °C for at least 6 h to obtain ZnO powder of various types with different aspect ratios, i.e., needle type, planar type, and vertical wall type. The commercially sold nanosized formless ZnO powder (SBC 30 N, SBC Co., Ansan, Republic of Korea) fabricated by wet precipitation method was used to compare properties with the developed powder.

The crystalline structure of the fabricated powder and its particle shape were examined using X-ray diffraction analysis (XRD, SmartLab, Rigaku Co., Tokyo, Japan) and scanning electron microscope (SEM, NOVA NANO 450, FEI Co., Hillsboro, OR, USA), respectively. The particle size distribution was measured using a particle size analyzer (PSA, Mastersizer 2000, Malvern Panalytical, Malvern, UK), while ultrasonic dispersion was performed for 1 min. The optical absorption of the fabricated powder post-processed using a counter jet mill (400AFG, Hosokawa Micron Co., Hirakata, Japan) was measured using the spectrometer (LAMBDA 950, PerkinElmer, Waltham, MA, USA) with a measurement range of 200–800 nm of UV light to the near-infrared wavelength.

### 2.2. Formulation and Characterization of Cosmetics Using the Prepared Powder

Cosmetics were formulated using lipophilic emulsifying agent as a medium so that the silicone oil was surrounded by the water molecules, and a W/S (water in silicone) formulation was prepared. The composition ratios for the test samples are shown in Table 2. Light absorption and transmission rate were compared for each type of ZnO. In this study, various types of ZnO powder at 16.1 wt.% composition were mixed, with the remaining components consisting of oil, water, and additives, etc. Triethoxycaprylyl silane (Dowcorning, Midland, MI, USA) was coated on the surfaces of the organic powder to add lipophilic properties before the formation of the cosmetic. To fabricate the internal oil phase, cyclopentasiloxane, PEG-10 dimethicone (BENTONEMULE WO, Ilion Korea, Seoul, Republic of Korea), emulsifier PEG/PPG-18/18 dimethicone (ES-5226 DM Formulation Aid, Dowcorning, USA), dispersant lauryl polyglyceryl-3 polydimethylsiloxyethyl dimethicone (KF-6115, Shinetsu, Tokyo, Japan), polyglyceryl-2 dipolyhydroxystearate (Dehymuls PGPH, Shinetsu, Japan) and oil-based cyclopentasiloxane, cyclohexasiloxane (PMX-0345, Dowcorning, USA), phenyl trimethicone (Dowcorning 556, Dowcorning, USA), dimethicone (PMX-2000 Silicone Fluid 10CS, Dowcorning, USA), hydrogenated poly (C6-14 Olefin) (Aloxsyn-4S, THE SHINE, Uiwang, Republic of Korea) were used. ZnO powder as the internal oil phase, commercial ZnO powder with particle sizes of 10–100 nm, and calamine powder were mixed at a set quantity and dispersed for approximately 1 h to make slurry samples. Lastly, dipropylene glycol (DPGFG, Goldleben, Cheongju, Republic of Korea), 2,3-butanediol (Greendiol, GS Caltex, Seoul, Republic of Korea), and glycerin (Cosnet, Yecheon, Republic of Korea) were added to the previously dispersed powder made as an internal oil phase in the purified water, and the cosmetic was fabricated via emulsification at 3000 rpm.

The formulated sample was spread thinly on the PMMA specimen, applied uniformly, dried at room temperature for 30 min or more, and then measured for optical transmittance using a spectrometer. For the in vivo test of the prepared formulation, the sample was applied to the test site at 2.0 mg/cm^2^, and then irradiated with ultraviolet rays using a Multiport solar simulator (Model 601 v.2.5, Solar Light, Glenside, PA, USA). Sun Protection Factor (SPF) and Protection grade of UVA (PA) were, respectively, determined according to the International organization for standardization, ISO 24444 and ISO 24442, by the Korea Dermatology Research Institute (KDRI), an international standards organization specializing in cosmetic application testing.

## 3. Results and Discussion

**Figure 2:**
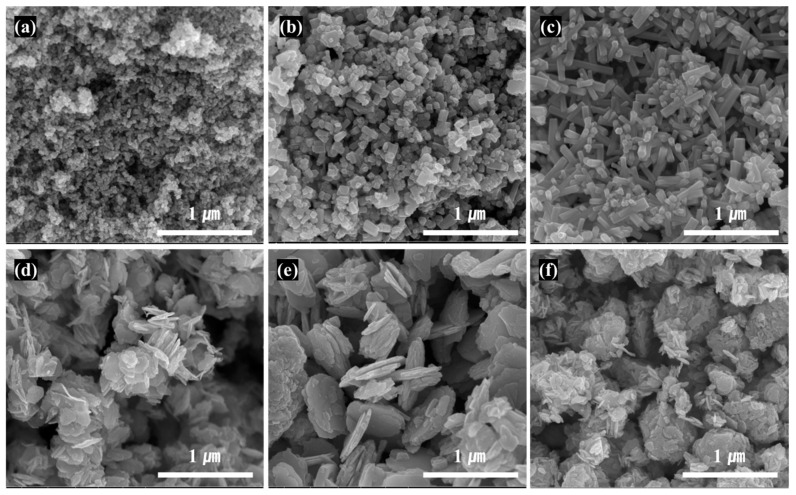
FE-SEM images of ZnO powder with different shapes: (**a**) Commercial Nano-sized ZnO, (**b**) LAR Needle-type ZnO, (**c**) HAR Needle-type ZnO, (**d**) LAR Planar-type ZnO, (**e**) HAR Planar-type ZnO and (**f**) Planar-type ZnO with vertical walls.

SEM images of ZnO particles with different types and aspect ratios made using various starting material and fabrication conditions are shown in Figure 2, and the summary on the morphology and size of the different ZnO particles is shown in Table 3. The particle shapes with a spherical particle with a size of 30 nm in (a), a short and thick needle shape in (b), a relatively thin and long needle shape in (c), a small hexagonal planar shape in (d), a large planar shape in (e), and a clustered shape in which planar particles are aggregated and grown in the thickness direction in (f) were observed in the SEM images of Figure 2. With zinc chloride as the starting ZnO salt, the SEM image indicated that the ZnO particles with needle shapes were all grown along the same direction. In general, the crystalline growth rate (v) was ranked as v[0001] ≫ v[01$\bar{1}$0] ≫ v[000$\bar{1}$] due to the ZnO crystalline characteristics; hence, needle-type ZnO with anisotropic needles along [0001] were synthesized [24,25]. When KOH concentration was low, small and short LAR (low aspect ratio) needle-type ZnO of length 100 nm, thickness 50 nm, and aspect ratio 2 were formed; when KOH concentration was as high as 20%, thin and long HAR (high aspect ratio) needle-type ZnO of length 400 nm, thickness 50 nm, and aspect ratio 8 were formed. Under low concentration, the rate of nucleus generation was slower, leading to less agglomeration of nucleus and formation of smaller particle sizes [26,27].

When zinc acetate was used as a starting Zn salt with added sodium citrate, hexagonal planar-type ZnO particles with similar particle sizes were formed. The growth of crystals along (01$\bar{1}$0) and perpendicular to (0001) was promoted, as the crystal growth along [0001] was suppressed due to the protection of the (0001) face of ZnO crystalline by anion citrate [28,29]. Using the input speed and KOH concentration shown previously, the LAR planar-type ZnO particle was grown along the width direction, and a HAR planar-type ZnO particle was approximately 200–700 nm in length and 15–30 nm in thickness. When the input speed of the KOH into the batch reactor was lowered (Table 1), HAR planar-type ZnO particles with a large size were formed, because as the input speed was lowered, it took longer to form the nucleus of zinc oxide, resulting in larger particle size [30,31]. Lastly, when the zinc chloride was used as a Zn salt, and sodium citrate was added, vertical wall-type ZnO was formed as planar-type particles were periodically arranged perpendicular to the surface of thin planar-type primary particles larger than 100 nm. In general, zinc chloride has faster reaction speed than zinc acetate, resulting in faster formation of particles, and with an increase in KOH concentration, the disassembly of Zn(OH)_4_^2−^ ions are accelerated, facilitating the growth of vertical partition walls due to easier formation of crystal seeds on top of the (000$\bar{1}$) surface [32,33]. Thin planar-type particles are singular particles that possess non-nano particle size in two directions, but vertical wall-type ZnO has lattices greater than 100 nm on all sides, confirming that all three directions had non-nano sizes. In Table 3, the large D50 (average particle size) value of commercial nanosized ZnO was due to the formation of secondary particles from agglomeration of primary particles.

**Figure 3:**
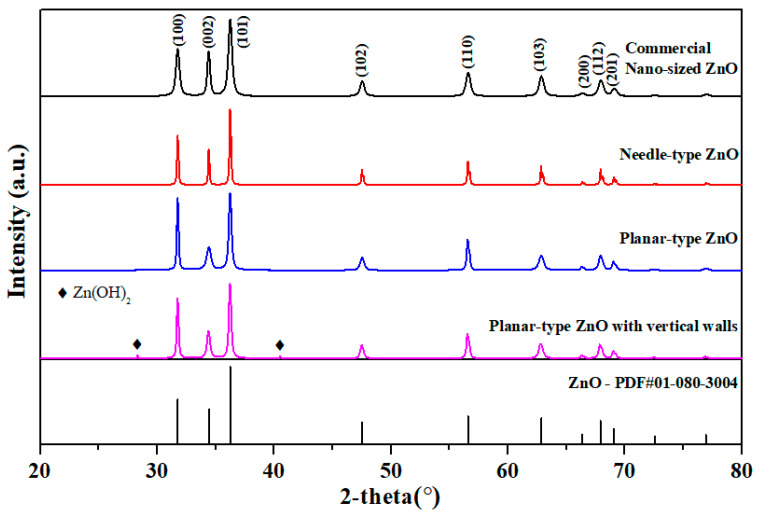
X-ray diffraction analysis of ZnO powder with different particle types.

The commercially sold nanosized formless ZnO powder and the prepared needle-type, planar-type, and vertical walls-type ZnO powder were compared using XRD analysis (Figure 3). The peak locations of all samples for ZnO were (100), (002), (101), (102) and (110). The (100) peak was higher than the (002) peak, causing easier removal of a reaction intermediate Zn(OH)_2_ and thus forming a single peak of pure ZnO with wurtzite structure without any formation of other impurities [34,35]. Some Zn(OH)_2_ was not removed in the vertical-type ZnO. Compared to nanosized commercial ZnO powder, the peaks of the fabricated powder were sharper, indicating the synthesis of superior crystallinity of ZnO.

**Figure 4:**
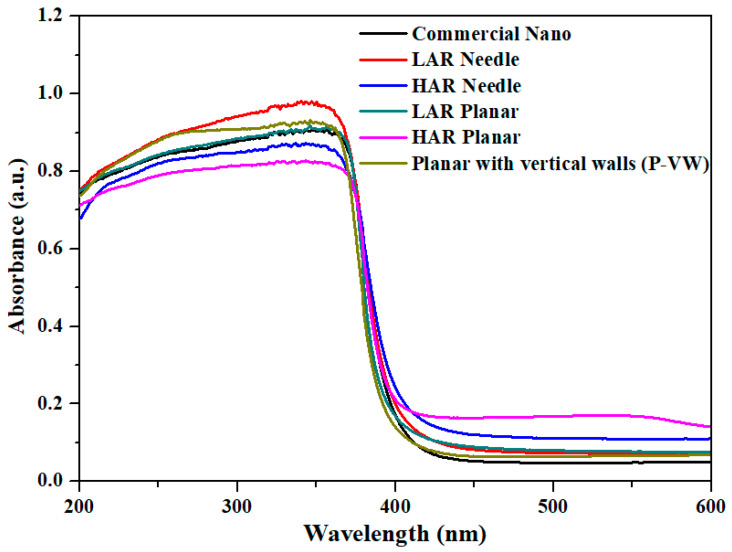
Light absorption spectrum for different types of ZnO powder.

Figure 4 shows the light absorption result in the 200–600 nm range of nanosized commercial ZnO powder and fabricated ZnO powder in different types. Considering the absorption in the UV light range, the maximum value of LAR needle-type was 98.1%, showing the highest light absorption, whereas for the HAR planar-type, it was 82.7%, indicating the lowest light absorption. In Table 3, when comparing the D50 (i.e., average particle size at cumulant particle size of 50%) values, the LAR needle type was 0.13 µm, the lowest value, and HAR planar type was 0.3 µm, a relatively high value. In general, the light absorption depends on the atoms reacting with the light path. As the particle size decreased, the interaction from more particles enabled higher absorption of UV light. Thus, the ZnO with smaller particle size showed better absorption performance due to higher particle count for the same weight [36,37]. Moreover, compared to the planar type, the vertical wall-type particles had light absorption of 93.2%, as the light could be absorbed multiple times due to the reflection on the (0001) faces [38,39]. The optical band gap (E_g_) of the powder calculated from Figure 4 was 3.06 to 3.15 eV, smaller than that of bulk ZnO (E_g_~3.37 eV). The energy band gap is size dependent and decreases with an increase in the crystallite size of the ZnO nanostructure due to the optical confinement effect. This behavior of E_g_ could also be explained based on surface-related defects and narrowing bandgap induced by oxygen vacancies in the synthesized powder [40,41].

**Figure 5:**
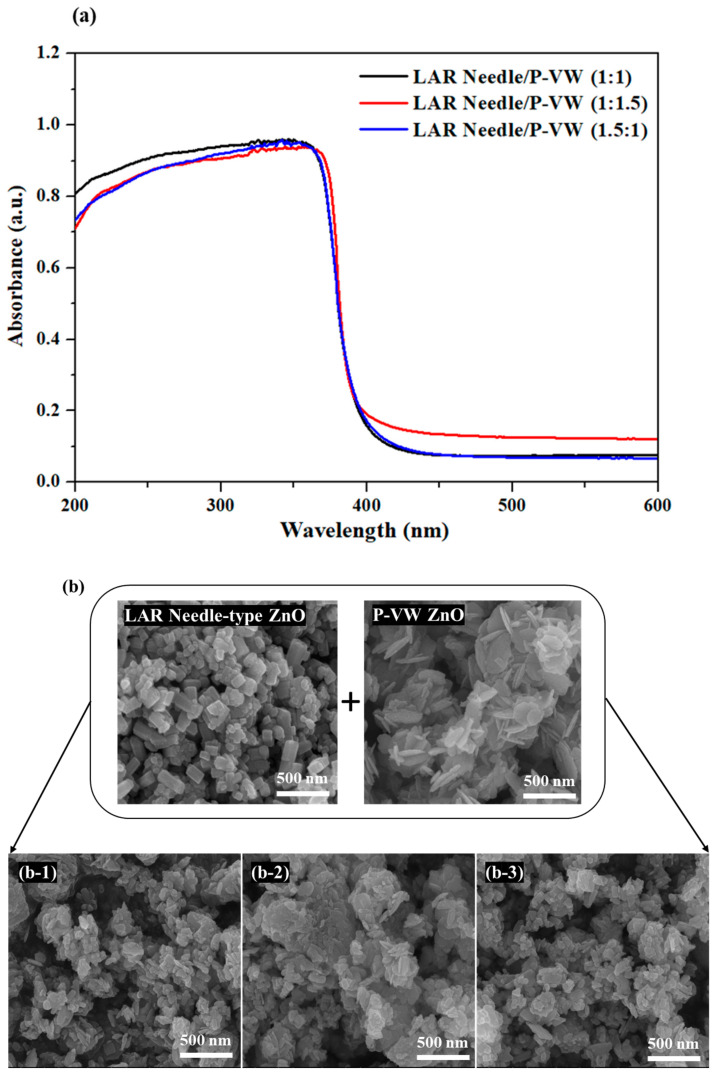
(**a**) Light absorption spectrum, (**b**) FE-SEM images of LAR needle and P-VW mixed powder at different ratios: (**b-1**) 1:1, (**b-2**) 1:1.5 and (**b-3**) 1.5:1.

Among the commercial ZnO powder with common nano sizes, the powder consisting of different shapes of nanosized particles displayed higher light absorption than nano powder with a single particle shape; hence, this study aimed to obtain higher UV light blockage by mixing different particle shapes of nanosized ZnO powder. The LAR needle-type ZnO, which showed the highest light absorption, and vertical wall-type ZnO, which was non-nanosized in 3D, were mixed into a cream sample. The light absorption and transmission of the cream sample were measured and compared with those of other products. Figure 5 displays the SEM images and light absorption of powders mixed by different ratios of LAR needle-type ZnO and vertical wall-type ZnO particles. The light absorption in the UV light range, shown in Figure 5a, is as high as 96% for ZnO powder mixture ratio of 1:1, and the mixture with 1:1.5 ratio of vertical wall-type ZnO to LAR needle-type ZnO exhibited the lowest absorption. Due to the even distribution of LAR needle-type particles between the vertical-type particles in the 1:1 powder mixture, as shown in the SEM image in Figure 5b, the light absorption was high. While the powder with LAR needle-type and the vertical wall-type particles all showed at least a 94% absorption rate, the 1:1.5 and 1.5:1 ratio mixture demonstrated lower absorption than the 1:1 ratio mixture, as singular particles were agglomerated on certain parts for mixtures with 1:1.5 or 1.5:1 ratios.

**Figure 6:**
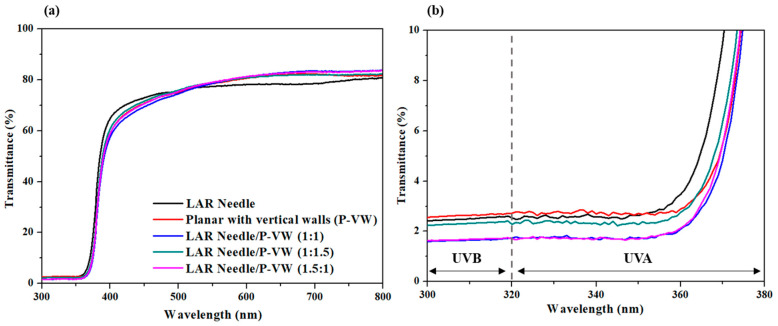
Light transmission spectrum for different types and mixtures of ZnO particles: (**a**) 300–800 nm wavelength, (**b**) 300–380 nm wavelength.

Figure 6 showed the results of light transmission of LAR needle-type ZnO, vertical wall-type ZnO, and a mixture of the two types made into cosmetic form. LAR needle-type and vertical wall-type ZnO showed relatively low light transmission of 2.5% and 2.6%, respectively. When powders with various dimensions are mixed, a decrease in transmittance is expected to occur due to the influence of Rayleigh and Mie scattering [42,43]. Rayleigh scattering is generated by particles smaller than the wavelength of incident light, while Mie scattering can occur in particles having a size similar to the wavelength. When at a 1:1 ratio, the light transmission at UVB and UVA regions were 1.7% and 3.8%, respectively, showing better UV blockage than products with singular shapes. This may be due to the maximization of light scattering and prevention of over-agglomeration of particles by evenly distributing LAR needle-type particles with vertical wall-type particles [44]. Moreover, LAR needle-type ZnO, compared to the mixed powder, exhibited high light absorption, while the light transmission for the 1:1 mixture in the UV region was 3.3%, which was the lowest value. At a 550 nm wavelength, the 1:1 mixture powder displayed the highest UV transmission, indicating that the functional group absorbed on the ZnO surface was well adsorbed on the dispersant, improving the dispersibility and preventing the agglomeration between particles, ultimately improving UV blocking abilities [45]. In an in vivo experiment on the mixture powder with a 1:1 ratio of LAR needle-type and vertical wall-type ZnO, the UV blockage efficacy was PA (Protection Grade of UVA) 8.2 ± 1.2 (PA+++) based on SPF (Sun Protection Factor) 40.3 ± 2.6 (SPF40), indicting a nano size ZnO level of UV blocking [46,47].

## 4. Conclusions

In this study, various types of non-nanosized ZnO powder were synthesized using the wet techniques outlined in the paper. The physical properties and the UV blockage efficacy of W/S-type sunscreens were evaluated. For LAR needle-type ZnO powder, high rates of light absorption (98.1%) and low light transmittance (5.4%) in the UV light region were observed, displaying superior light blockage effect (2.5%) in the UVB area. The vertical wall-type ZnO powder also displayed high light absorption (93.2%) and low light transmission (4.1%), in particular, low light transmission (4.6%) in the UVA region when compared to LAR needle type (6.3%). The light transmission of a cosmetic product with a 1:1 ratio of LAR needle-type ZnO with highest light absorption and vertical wall-type ZnO powder that was non-nanosized in all dimensions was 1.7% and 3.8% in UVB and UVA regions, respectively. Compared to other cosmetics formulated with LAR needle or P-VW powder singly, this mixed cosmetic exhibited a superior light-blocking effect by improved dispersibility and prevention of particle agglomeration. The 1:1 mixed sample had non-nanosized particles, thus complying with the European nanomaterials regulation. Moreover, the 1:1 mixed sample displayed high light absorption and low light transmission compared to generic ZnO nanoparticles. In addition, with superior UV protection in the UVA and UVB regions, the 1:1 mixed powder showed potential to be used as a main ingredient in UV protection cosmetics.

## Figures and Tables

**Table 1:** Starting materials and KOH usage conditions for making differently shaped ZnO powder.

	Batch Reactor	KOH	
		Concentration (%)	Feeding Rate (g/min)
LAR ^(1)^-Needle-type ZnO	Zinc chloride	15	130
HAR ^(2)^-Needle-type ZnO		20	140
LAR-Planar-type ZnO	Zinc acetate + Sodium citrate	5~6	2000
HAR-Planar-type ZnO		5~6	800
Planar-type ZnO with vertical walls	Zinc chloride + Sodium citrate	15	160

^(1)^ Low Aspect Ratio, ^(2)^ High Aspect Ratio.

**Table 2:** Composition of cosmetic W/S type materials.

Phases	Materials	Composition (wt%)
Powder	ZnO	16.1
	ZnO(nano)	5.1
	Calamine	3.8
Oil	Cyclopentasiloxane, PEG-10 Dimethicone	2
	PEG/PPG-18/18 Dimethicone	3
	Lauryl polyglyceryl-3 polydimethylsiloxyethyl dimethicone	1
	Polyglyceryl-2 Dipolyhydroxystearate	1
	Cyclopentasiloxane, Cyclohexasiloxane	11
	Phenyl Trimethicone	8.5
	Dimethicone	0.5
	Hydrogenated Poly(C6-14 Olefin)	3
Water	Dipropylene Glycol	2
	2,3-Butanediol	2
	Glycerin 99.5%	1
	Water	40
Total		100

**Table 3:** Summary on morphology and sizes of differently shaped ZnO particles.

	Length (nm)	Thickness (nm)	Aspect Ratio	# of Non-Nano Dimension	D50 (µm)
Nano-sized ZnO	30	30	1	None	1.48
LAR-Needle-type ZnO	100	50	2	1	0.13
HAR-Needle-type ZnO	400	50	8	1	0.24
LAR-Planar-type ZnO	200	15	13	2	0.23
HAR-Planar-type ZnO	700	30	23	2	0.36
Planar-type ZnO with vertical walls	150	20	7.5	3	0.15

## Data Availability

The data presented in this study are contained within the article.
